# Retrospective analysis of intestinal flora alterations in adults with acute gastrointestinal injury

**DOI:** 10.3389/fcimb.2026.1776653

**Published:** 2026-06-05

**Authors:** Zhijian Jiang, Aijie Tang, Min Zhao, Yifan Long, Yongguo Cai

**Affiliations:** 1 Department of Emergency Medicine, China Coast Guard Hospital, Jiaxing, Zhejiang, China; 2 Department of gastroenterology, 970 Hospital of the Joint Logistics Support Force of the People’s Liberation Army (PLA), Yantai, Shandong, China

**Keywords:** acute gastrointestinal injury, gut microbiota, ICU outcomes, intestinal dysbiosis, microbial diversity

## Abstract

**Background:**

Acute Gastrointestinal Injury is a common and serious complication in critically ill adults associated with poor outcomes. Its progression involves intestinal barrier dysfunction, nutritional impairment, and gut microbiota alterations, which remain inadequately characterized across severity grades.

**Objective:**

This study aimed to evaluate the association between gut microbiota composition, nutritional status, clinical severity, and outcomes.

**Methods:**

A retrospective cross-sectional study of 550 adults with AGI (grades I–IV) and controls was conducted across multiple tertiary hospitals from June to September 2025, including 343 males. Clinical, nutritional, biochemical, and microbiota data were analyzed by AGI severity using diversity measures and multivariate regression models.

**Results:**

Increasing AGI severity was associated with age, higher comorbidity burden, greater illness severity, increased organ support requirements, and worse clinical outcomes, including prolonged ICU stay and higher mortality (p < 0.001). Severe AGI (III–IV) patients showed pronounced gastrointestinal dysfunction, delayed and interrupted enteral nutrition, reduced caloric adequacy, and significantly impaired nutritional biomarkers. Marked gut dysbiosis was observed with increasing AGI severity, characterized by significantly reduced microbial diversity, depletion of beneficial taxa (*Firmicutes, Bacteroidetes, Lactobacillus, Bifidobacterium, Faecalibacterium*), and overgrowth of potentially pathogenic bacteria, particularly Proteobacteria and Escherichia–Shigella (all p < 0.001). Multivariate analysis identified higher age, APACHE II and SOFA scores, antibiotic and proton pump inhibitor use, and interruption of enteral nutrition as independent risk factors associated with severe AGI and dysbiosis, whereas higher BMI, serum albumin levels, and greater microbial diversity were associated with a lower likelihood of severe AGI.

**Conclusion:**

AGI severity is closely associated with worsening intestinal dysbiosis, deteriorating nutritional status, and poorer clinical outcomes. These findings highlight the importance of early nutritional support and microbiota-targeted interventions in managing critically ill AGI patients.

## 1 Introduction

Acute gastrointestinal injury (AGI) is the main cause of death among critically ill patients and a condition that co, exists most of the time in these patients (Zhang et al., 2023). It is defined as gastrointestinal tract dysfunction and damage of the mucosal lining along with systemic inflammatory reactions (Cortese et al., 2021; Di Vincenzo et al., 2024). The gut microbiota consists of bacteria, fungi, and other microorganisms that live in the gut and are responsible for the intestinal barrier function as well as immune regulation (Colella et al., 2023). They also change the metabolism of nutrients. The latest results indicate that AGI is a major factor that leads to changes in intestinal flora; more often than not, these changes are characterized by lower microbial diversity, reduced levels of commensal organisms such as *Lactobacillus*, *Bifidobacterium*, and *Faecalibacterium*, and increased levels of opportunistic pathogens such as *Escherichia Shigella*, *Enterococcus*, and *Klebsiella* (Su and Ding, 2023). These dysbiotic changes result in compromised gut barrier integrity, consequently leading to a raised intestinal permeability level (Stolfi et al., 2022), bacterial translocation, and systemic inflammatory response exacerbation that can lead to multi-organ failure development. On the level of mechanisms, the changes in the microbiota and metabolic disturbances are caused by hypoperfusion related to critical illness (Owen et al., 2022), the presence of systemic inflammatory mediators, administration of broad-spectrum antibiotics, use of proton pump inhibitors (Giordan et al., 2021), and reduction of enteral nutrition. Understanding patterns and reasons of microbial changes in the gut in AGI is a prerequisite for designing targeted interventions (Gao et al., 2025) that include the use of microbiota, modulating drugs to restore gut homeostasis, and improve clinical outcomes in this vulnerable patient population (Cortés et al., 2025). Clinically, it is divided into four categories (I to IV) based on the extent of the damage, a temporary risk of dysfunction (Grade I) to a risk of death due to gastrointestinal failure (Grade IV), as well as the chance of progression to multiple organ dysfunction syndrome (MODS) and shock (Zhong et al., 2021; Gallo et al., 2024). This grading system follows the internationally recognized criteria proposed by the European Society of Intensive Care Medicine (ESICM) Working Group on Abdominal Problems, where Grade I indicates increased risk of GI dysfunction (e.g., mild symptoms such as nausea or reduced intake), Grade II represents GI dysfunction requiring interventions (e.g., feeding intolerance, gastric residuals), Grade III reflects GI failure with persistent intolerance despite treatment, and Grade IV denotes life-threatening GI failure associated with severe complications such as ischemia, abdominal compartment syndrome, or perforation. It is caused by a combination of variables, including splanchnic hypoperfusion, ischemia–reperfusion injury (Bourcier et al., 2021), systemic inflammation, and neuroendocrine dysregulation, which affect the movement of the intestines, the blood supply to the mucosa, and the integrity of the epithelium (Docsa et al., 2022). These changes in the body’s physiology weaken the gut barrier, making the intestines more permeable (Linares et al., 2021). This allows microbes to move freely, triggering the body’s immunological responses, which exacerbate local and systemic inflammation (Di Tommaso et al., 2021). Loss of gut barrier function not only hinders digestion and absorption but also promotes the translocation of luminal bacteria and endotoxins into the bloodstream (Soranno et al., 2025), increasing the risk of sepsis and multiple organ dysfunction syndrome (MODS). The GI tract is very important for immune regulation and homeostasis (AbdulRaheem, 2023), thus an acute injury to it makes clinical outcomes in critical illness much worse (McLean et al., 2023). This shows how important it is to recognize and treat these injuries quickly (Yeh et al., 2022). Despite an increasing understanding that the gut microbiota plays a major role in critical disease, there is limited information from clinical studies, especially retrospective ones, on how the gut microbiota differs in adult patients with acute gastrointestinal illness (AGI). This study was undertaken to address the limited understanding of how intestinal microbiota alterations correlate with disease severity, nutritional deterioration, and outcomes in adults with acute gastrointestinal injury. Clarifying these relationships may support earlier risk stratification and targeted nutritional and microbiota-based interventions in critically ill patients.

## 2 Material and method

### 2.1 Study design and setting

A retrospective cross-sectional study was performed to observe changes in intestinal flora in adult patients with acute gastrointestinal injury (AGI). Data were extracted from patients’ file records, including medical records, computerized databases, and microbial profile results from a tertiary care hospital, from June to September 2025. The study sought to evaluate the makeup of gut microbiota, clinical severity, nutritional status, and related parameters in adults with differing grades of acute gastrointestinal infection (AGI).

### 2.2 Study population and sample size

The participants were selected using a convenience sampling approach, whereby patient records readily available in hospital intensive care units (ICUs) and electronic health databases were included. Specifically, data were extracted from accessible hospital files within the study period rather than through random selection, which may introduce selection bias and limit the generalizability of findings. Inclusion criteria comprised adults aged 18 years and above with a confirmed diagnosis of AGI (grades I–IV) and complete clinical, laboratory, and microbiome data. Patients with long-term gastrointestinal diseases (e.g., inflammatory bowel disease), those who had undergone gastrointestinal surgery in the last 3 months, severely immunocompromised individuals, and those with incomplete medical records were excluded from the study.

### 2.3 Data collection tool and procedure

We used a structured data abstraction form to pull out data after looking at published research on AGI, gut flora, and critical care nutrition. The collected data was from four areas: Sociodemographic traits: age, gender, body mass index (BMI), and concomitant conditions. Clinical characteristics: AGI grade, illness severity scores (APACHE II, SOFA), ICU admission, organ support (mechanical breathing, vasopressors, renal replacement treatment), and biochemical indicators (serum albumin, prealbumin, citrulline, I-FABP). Management and nutritional parameters: enteral and parenteral nutrition practices, caloric sufficiency, and drug exposure (antibiotics, proton pump inhibitors). Assessment of intestinal microbiota: alpha and beta diversity indices (Observed OTUs, Chao1, ACE, Shannon, Simpson), relative abundance of dominant bacterial phyla (*Firmicutes*, *Bacteroidetes*, *Proteobacteria*, *Actinobacteria*, *Fusobacteria*), and essential genera (*Escherichia–Shigella*, *Enterococcus*, *Klebsiella*, *Lactobacillus*, *Bifidobacterium*, *Faecalibacterium*).

### 2.4 Ethical considerations

This research was approved by the Ethics Committee of 970 Hospital of the Joint Logistics Support Force of the PLA, No.20251201. The study complied with the Declaration of Helsinki. Since this was a retrospective review, informed consent was not needed, however patient privacy and data privacy were scrupulously protected.

### 2.5 Statistical analysis

We used SPSS version 26 to analyze the data. Descriptive statistics included a summary of the patients’ demographics, clinical characteristics, dietary factors, microbiota composition, and therapy information. We showed continuous variables as mean ± standard deviation (SD) and categorical variables as counts and percentages. We used chi-square tests for categorical variables and ANOVA for continuous variables to compare groups based on how bad their AGI was. We used logistic regression and multivariate linear regression to identify clinical and microbiota-related factors associated with AGI severity and gut microbiota alterations. To reduce potential confounding, multivariate models were adjusted for major demographic and clinical covariates, including age, sex, and APACHE II score, together with other clinically relevant variables. A p-value less than 0.05 was seen to be statistically significant.

## 3 Results

### 3.1 Expanded baseline demographic, clinical severity, treatment, and outcomes of the study population

**Table 1** shows that patients with more severe acute gastrointestinal injury (AGI) had very different demographic, clinical, therapeutic, and outcome features than patients with less severe disease and controls. The mean age exhibited a significant increase across groups (p = 0.002), with AGI III–IV patients being older (59.9 ± 14.2 years) compared to those with AGI I–II (56.8 ± 13.4 years) and controls (54.2 ± 12.6 years). In contrast, the sex distribution was similar among groups (male: 66.1%, 61.8%, and 58.7%, respectively; p = 0.28). The body mass index was considerably lower in AGI III–IV patients (22.9 ± 3.4 kg/m²) compared to AGI I–II (23.7 ± 3.1 kg/m²) and controls (24.2 ± 3.0 kg/m²; p = 0.01), indicating potential disease-related catabolism or nutritional deficiency. The prevalence of comorbidities escalated with the severity of AGI, with hypertension affecting 43.9% of AGI III–IV patients compared to 38.2% in AGI I–II and 27.3% in controls (p = 0.01), diabetes mellitus in 34.4% versus 26.8% and 19.3% (p = 0.02), and chronic kidney disease in 26.7% versus 14.1% and 8.0%, respectively (p < 0.001). There was a clear stepwise rise in clinical severity scores across groups. For example, APACHE II scores were 22.8 ± 5.6 in AGI III–IV patients, 14.6 ± 4.3 in AGI I–II patients, and 9.1 ± 3.2 in controls. SOFA scores were 10.4 ± 3.3, 6.3 ± 2.1, and 3.2 ± 1.4, respectively (both p < 0.001). All patients with AGI need ICU hospitalization, but merely 41.3% of controls were admitted (p < 0.001). The need for organ support therapies rose significantly with AGI severity, including mechanical ventilation (76.1% in AGI III–IV vs. 40.0% in AGI I–II and 14.0% in controls), vasopressor use (71.7% vs. 32.3% and 12.0%), and renal replacement therapy (27.2% vs. 8.2% and 2.7%) (all p < 0.001). Patients with AGI III–IV were far more likely to have taken antibiotics in the last three months (84.4%) than those with AGI I–II (67.7%) or controls (28.7%). Proton pump inhibitors were also used more often in AGI III–IV patients (81.1%, 59.5%, and 34.7%, respectively; both p < 0.001). As AGI severity increased, clinical outcomes got worse. For example, ICU stays were longer (15.8 ± 6.3 days in AGI III–IV vs. 9.6 ± 4.1 days in AGI I–II and 6.2 ± 2.8 days in controls) and hospital stays were longer (24.9 ± 9.1 vs. 16.2 ± 6.7 and 11.4 ± 4.9 days; p < 0.001). Mortality exhibited a distinct severity-dependent gradient, with ICU mortality rates of 32.2% in AGI III–IV patients, contrasted with 9.5% in AGI I–II and 3.3% in controls, and 28-day mortality rates of 39.4%, 12.3%, and 4.7%, respectively (both p < 0.001). Overall, these findings show that as AGI severity goes up, so does age, comorbidity burden, disease severity, treatment needs, and short-term clinical outcomes.

**Table 1:** Expanded baseline demographic, clinical severity, treatment, and outcomes of the study population.

Variable (N = 550)	AGI I–II (n=220)	AGI III–IV (n=180)	Control (n=150)	p-value
Demographics				
Age (years), mean ± SD	56.8 ± 13.4	59.9 ± 14.2	54.2 ± 12.6	0.002
Male sex, n (%)	136 (61.8)	119 (66.1)	88 (58.7)	0.28
BMI (kg/m²)	23.7 ± 3.1	22.9 ± 3.4	24.2 ± 3.0	0.01
Comorbidities				
Hypertension, n (%)	84 (38.2)	79 (43.9)	41 (27.3)	0.01
Diabetes mellitus, n (%)	59 (26.8)	62 (34.4)	29 (19.3)	0.02
Chronic kidney disease, n (%)	31 (14.1)	48 (26.7)	12 (8.0)	<0.001
Severity Scores				
APACHE II score	14.6 ± 4.3	22.8 ± 5.6	9.1 ± 3.2	<0.001
SOFA score	6.3 ± 2.1	10.4 ± 3.3	3.2 ± 1.4	<0.001
ICU-Related Variables				
ICU admission, n (%)	220 (100)	180 (100)	62 (41.3)	<0.001
Mechanical ventilation, n (%)	88 (40.0)	137 (76.1)	21 (14.0)	<0.001
Vasopressor use, n (%)	71 (32.3)	129 (71.7)	18 (12.0)	<0.001
Renal replacement therapy, n (%)	18 (8.2)	49 (27.2)	4 (2.7)	<0.001
Medication Exposure				
Antibiotic exposure (≤3 months), n (%)	149 (67.7)	152 (84.4)	43 (28.7)	<0.001
Proton pump inhibitor use, n (%)	131 (59.5)	146 (81.1)	52 (34.7)	<0.001
Clinical Outcomes				
ICU length of stay (days)	9.6 ± 4.1	15.8 ± 6.3	6.2 ± 2.8	<0.001
Hospital length of stay (days)	16.2 ± 6.7	24.9 ± 9.1	11.4 ± 4.9	<0.001
ICU mortality, n (%)	21 (9.5)	58 (32.2)	5 (3.3)	<0.001
28-day mortality, n (%)	27 (12.3)	71 (39.4)	7 (4.7)	<0.001

Data are presented as mean ± standard deviation for continuous variables and number (%) for categorical variables. Comparisons among AGI I–II, AGI III–IV, and control groups were performed using one-way ANOVA for continuous variables and χ² test for categorical variables, as appropriate. A p-value < 0.05 was considered statistically significant.

### 3.2 Integrated acute gastrointestinal injury features, nutritional status, and intestinal microbiota characteristics

**Table 2** shows that data extracted from patient records indicate that individuals with acute gastrointestinal injury (AGI) had significantly different outcomes, varying with severity, based on their gastrointestinal symptoms, nutritional status, and intestinal microbiota composition compared to the control group. The occurrence of clinical symptoms of gastrointestinal dysfunction was significantly higher in AGI III–IV patients, whereas only 35.5% of AGI III and 4.0% of controls experienced it (p < 0.001). Besides this, very high gastric residual volumes (>500 mL/day) were found in 72.8% of patients with severe AGI, while only 29.1% of those with mild to moderate AGI and 3.3% of controls had this condition (p < 0.001). Accordingly, the proportion of diarrhea in patients with AGI III–IV was 65.6% as compared to 31.4% of AGI III and 9.3% of controls. The numbers of patients with abdominal distension were 82.8%, 37.3%, and 6.0%, respectively (all p < 0.001). More serious problems, like gastrointestinal hemorrhage (28.3%) and paralytic ileus (26.1%), were mostly seen in AGI III–IV patients. This shows that gastrointestinal dysfunction gets worse as AGI gets worse. Patients with AGI had a lot of trouble with their gastrointestinal function and nutritional factors. The worst problems were seen in AGI III–IV. The time to the start of enteral nutrition was much longer in severe AGI (4.6 ± 1.8 days) than in AGI I–II (2.1 ± 0.9 days) and controls (1.2 ± 0.4 days; p < 0.001). Also, enteral nutrition was stopped more often in AGI III–IV patients (60.6%) than in AGI I–II (23.6%) and controls (5.3%). In severe AGI, dependency on parenteral nourishment was much greater (76.7%) than in AGI I–II (30.5%) and controls (7.3%) (p < 0.001). Caloric adequacy on day 7 diminished steadily with increasing AGI severity, achieving only 51.2 ± 14.3% in AGI III–IV patients, compared to 78.4 ± 12.6% in AGI I–II and 92.1 ± 8.9% in controls. Biochemical markers of nutritional and intestinal status corroborated this trend, revealing significantly reduced serum albumin (27.3 ± 3.8 g/L), prealbumin (118 ± 39 mg/L), and citrulline levels (13.4 ± 5.1 µmol/L), along with markedly elevated intestinal fatty acid–binding protein (I-FABP; 687 ± 164 pg/mL) in AGI III–IV patients, indicating severe mucosal injury and compromised absorptive capacity. A study of gut microbiota diversity showed that as AGI worsened, the number and variety of microbes in the stomach declined significantly. In patients with AGI, alpha diversity indices, such as observed operational taxonomic units (OTUs), Chao1, ACE, Shannon, and Simpson indices, were significantly lower, with the lowest values seen in AGI III–IV (e.g., Shannon index 2.71 ± 0.48) compared to AGI I–II (3.62 ± 0.54) and controls (4.28 ± 0.61; all p < 0.001). This shows that severe AGI is characterized by marked dysbiosis.

**Table 2:** Integrated acute gastrointestinal injury features, nutritional status, and intestinal microbiota characteristics. .

Domain (N = 550)	Parameter	AGI I–II (n=220)	AGI III–IV (n=180)	Control (n=150)	p-value
A. AGI Clinical Manifestations	Feeding intolerance, n (%)	78 (35.5)	142 (78.9)	6 (4.0)	<0.001
	Gastric residual volume >500 mL/day	64 (29.1)	131 (72.8)	5 (3.3)	<0.001
	Diarrhea (>3 stools/day)	69 (31.4)	118 (65.6)	14 (9.3)	<0.001
	Abdominal distension	82 (37.3)	149 (82.8)	9 (6.0)	<0.001
	Gastrointestinal bleeding	19 (8.6)	51 (28.3)	2 (1.3)	<0.001
	Paralytic ileus	14 (6.4)	47 (26.1)	0 (0.0)	<0.001
B. Gastrointestinal Function & Nutritional Parameters	Time to enteral nutrition (days), mean ± SD	2.1 ± 0.9	4.6 ± 1.8	1.2 ± 0.4	<0.001
	Enteral nutrition interruption, n (%)	52 (23.6)	109 (60.6)	8 (5.3)	<0.001
	Parenteral nutrition use, n (%)	67 (30.5)	138 (76.7)	11 (7.3)	<0.001
	Caloric adequacy at day 7 (%)	78.4 ± 12.6	51.2 ± 14.3	92.1 ± 8.9	<0.001
	Serum albumin (g/L)	32.6 ± 4.1	27.3 ± 3.8	38.2 ± 4.5	<0.001
	Prealbumin (mg/L)	182 ± 46	118 ± 39	241 ± 52	<0.001
	Citrulline (µmol/L)	21.8 ± 6.2	13.4 ± 5.1	29.6 ± 7.0	<0.001
	I-FABP (pg/mL)	412 ± 118	687 ± 164	215 ± 72	<0.001
C. Alpha Diversity of Gut Microbiota	Observed OTUs	312 ± 54	221 ± 47	386 ± 61	<0.001
	Chao1 index	368 ± 61	261 ± 58	442 ± 74	<0.001
	ACE index	351 ± 57	248 ± 51	427 ± 69	<0.001
	Shannon index	3.62 ± 0.54	2.71 ± 0.48	4.28± 0.61	<0.001
	Simpson index	0.78± 0.09	0.64± 0.11	0.86 ± 0.07	<0.001

Data are presented as mean ± standard deviation for continuous variables and number (%) for categorical variables. Comparisons among AGI I–II, AGI III–IV, and control groups were performed using one-way ANOVA for continuous variables and χ² test for categorical variables, as appropriate. A p-value < 0.05 was considered statistically significant.

**Figure 1:**
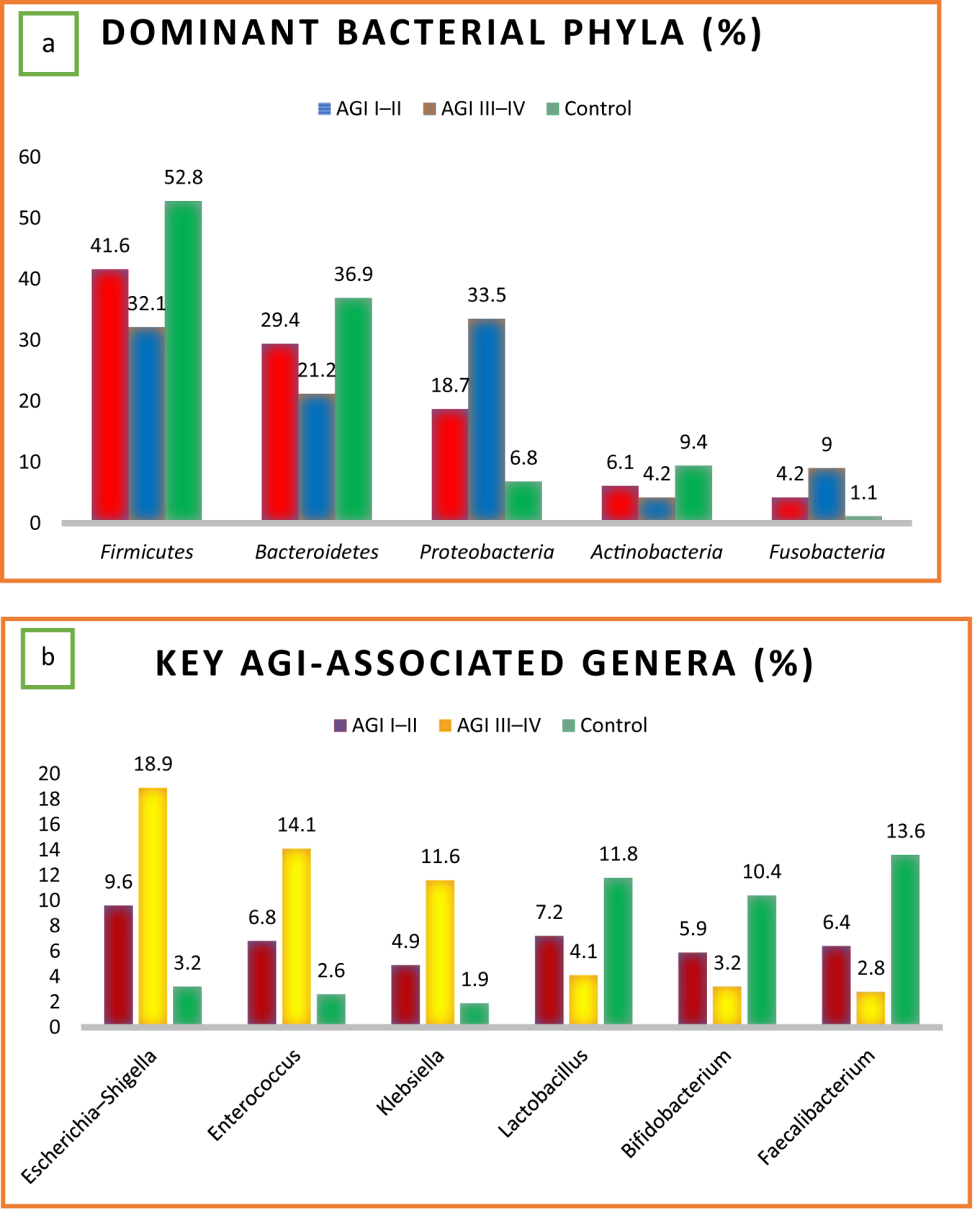
Integrated analysis of acute gastrointestinal injury features and intestinal microbiota characteristics **(a)** dominant bacterial phyla (%), **(b)** key AGI-associated genera (%).

At the phylum level, AGI was marked by a large decrease in beneficial *Firmicutes* and *Bacteroidetes*, along with a simultaneous increase in possibly harmful *Proteobacteria* and *Fusobacteria*. In AGI III–IV patients, Proteobacteria abundance increased to 33.5 ± 9.3%, compared to 18.7 ± 6.4% in AGI I–II patients and only 6.8 ± 3.1% in controls. Firmicutes and Bacteroidetes, on the other hand, decreased to 32.1 ± 8.7% and 21.2 ± 7.6%, respectively, in severe AGI (p < 0.001). At the genus level, severe AGI was linked to a higher number of opportunistic and possibly harmful taxa, such as *Escherichia–Shigella* (18.9 ± 6.8%), *Enterococcus* (14.1 ± 5.7%), and *Klebsiella* (11.6 ± 4.9%). In AGI I–II and controls, these taxa were much less common (all p < 0.001). Conversely, beneficial commensal genera with anti-inflammatory and barrier-protective roles, such as *Lactobacillus*, *Bifidobacterium*, and *Faecalibacterium*, were significantly diminished in AGI III–IV patients (4.1 ± 2.2%, 3.2 ± 1.9%, and 2.8 ± 1.6%, respectively) compared to AGI I–II and control groups, where these taxa were more prevalent (**Figures 1a, b**).

### 3.3 Multivariate regression analysis of clinical and microbiota factors with AGI severity and gut microbiota

To account for potential confounding between illness severity and treatment-related variables, adjusted multivariate analyses were performed, controlling for age, sex, and APACHE II score. After adjustment, the associations between antibiotic exposure, proton pump inhibitor use, interruption of enteral nutrition, serum albumin levels, and gut microbiota characteristics remained statistically significant, although several effect sizes were modestly attenuated. **Table 3** shows multivariate analysis that finds clinical and microbiota-related parameters that are independently linked to AGI severity and important gut microbiota traits in the study population (N = 550). As age increased, the chance of severe AGI rose by 4% for each additional year (OR = 1.04, 95% CI 1.01–1.07; p = 0.012). Age correlated with increased gut dysbiosis, indicated by a decrease in microbial diversity (Shannon index β = –0.012; p = 0.018), an elevation in Proteobacteria abundance (β = 0.15; p = 0.001), and a diminished *Firmicutes/Bacteroidetes* (F/B) ratio (β = –0.017; p = 0.009). There was no significant link between male sex and AGI severity or microbiome parameters (all p > 0.05). On the other hand, a high BMI was a protective factor against severe AGI (OR = 0.91 per kg/m; p = 0.004) and was positively correlated with gut ecology as shown by the increased Shannon diversity (β = 0.07; p = 0.001), lowered Proteobacteria levels (β = -0.9; p = 0.005) and increased F/B ratio (β = 0.09; p = 0.004). The severity pattern of the critically ill was well and deeply correlated. The rise of APACHE II score by one point was increasing the risk of severe AGI by 21% (OR = 1.21; p < 0.001) and was linked with a considerable decrease in microbial diversity (β = 0.08; p < 0.001), an increase in Proteobacteria abundance (β = 2.0; p < 0.001) and a decrease of the F/B ratio (β = 0.11; p < 0.001). Higher SOFA scores, similarly, showed a stronger association with severe AGI (OR = 1.35; p < 0.001) and severe dysbiosis that were characterized by the reduction of the Shannon index (β = 0.10), the increase of Proteobacteria count (β= 2.8), and the decrease of the F/B ratio (β = –0.14), all significantly at p < 0.001. Antibiotic treatment significantly elevated the probability of severe AGI (OR = 2.47; p = 0.001) and was correlated with a marked reduction in microbial diversity (β = –0.21), an increase in Proteobacteria (β = 4.5), and a diminished F/B ratio (β = –0.26). The use of proton pump inhibitors also doubled the likelihood of severe AGI (OR = 2.03; p = 0.008) and independently caused dysbiosis, as seen by a lower Shannon index (β = –0.17) and a higher Proteobacteria level (β = 3.2). Nutritional factors were some of the best predictors. Stopping enteral nutrition was linked to a more than threefold increase in the risk of severe AGI (OR = 3.12; p < 0.001) and the most severe microbial changes, such as a big drop in diversity (β = –0.26), a big rise in Proteobacteria (β = 5.8), and a big drop in the F/B ratio (β = –0.33). On the other hand, higher serum albumin levels were protective, lowering the risk of severe AGI by 12% for every g/L increase (OR = 0.88; p < 0.001). They were also strongly linked to higher microbial diversity (β = 0.12), lower Proteobacteria abundance (β = –1.8), and the restoration of the F/B ratio (β = 0.14). Gut microbiota indices were independent predictors of AGI severity: a higher Shannon index significantly decreased the risk of severe AGI (OR = 0.41; p < 0.001), while increased *Proteobacteria* (OR = 1.09 per 1% increase; p < 0.001) and *Escherichia–Shigella* abundance (OR = 1.12; p < 0.001) were strongly correlated with worsening AGI. These data collectively highlight the interconnected roles of illness severity, nutritional status, and gut microbial dysbiosis in the advancement of acute gastrointestinal damage.

**Table 3:** Multivariate regression analysis of clinical and microbiota factors with AGI severity and gut microbiota.

Variable	Logistic regression: severe AGI (OR, 95% CI)	p-value	Linear regression: Shannon index β (95% CI)	p-value	Linear regression: proteobacteria (%) β (95% CI)	p-value	Linear regression: F/B ratio β (95% CI)	p-value
Age (per year)	1.04 (1.01–1.07)	0.012	–0.012 (–0.022 to –0.002)	0.018	0.15 (0.06–0.24)	0.001	–0.017 (–0.030 to –0.004)	0.009
Male sex	1.18 (0.75–1.84)	0.47	–0.05 (–0.20 to 0.10)	0.52	1.2 (–0.4 to 2.8)	0.14	0.08 (–0.12 to 0.28)	0.44
BMI (kg/m²)	0.91 (0.85–0.97)	0.004	0.07 (0.03–0.12)	0.001	–0.9 (–1.5 to –0.3)	0.005	0.09 (0.03–0.15)	0.004
APACHE II score	1.21 (1.13–1.30)	<0.001	–0.08 (–0.12 to –0.05)	<0.001	2.0 (1.2–2.8)	<0.001	–0.11 (–0.16 to –0.06)	<0.001
SOFA score	1.35 (1.20–1.52)	<0.001	–0.10 (–0.15 to –0.06)	<0.001	2.8 (1.8–3.8)	<0.001	–0.14 (–0.21 to –0.07)	<0.001
Antibiotic exposure	2.47 (1.45–4.21)	0.001	–0.21 (–0.34 to –0.08)	0.002	4.5 (2.0–7.0)	0.001	–0.26 (–0.42 to –0.10)	0.002
Proton pump inhibitor use	2.03 (1.20–3.45)	0.008	–0.17 (–0.31 to –0.03)	0.016	3.2 (1.0–5.4)	0.004	–0.19 (–0.33 to –0.05)	0.009
Enteral nutrition interruption	3.12 (1.87–5.22)	<0.001	–0.26 (–0.39 to –0.13)	<0.001	5.8 (3.4–8.2)	<0.001	–0.33 (–0.49 to –0.17)	<0.001
Serum albumin (g/L)	0.88 (0.82–0.94)	<0.001	0.12 (0.07–0.17)	<0.001	–1.8 (–2.7 to –0.9)	<0.001	0.14 (0.06–0.22)	0.001

Logistic regression: Dependent variable = severe AGI (III–IV vs I–II), Linear regression: Dependent variables = Shannon index & Proteobacteria %, Continuous variables: β coefficients; Categorical variables: coded 0/1, p < 0.05 considered significant.

## 4 Discussion

The present investigation reveals that patients with elevated levels of acute gastrointestinal injury (AGI III–IV) exhibit significantly worse demographic, clinical, therapeutic, and prognostic profiles than those with lower-grade AGI (III) and control subjects. Our results are consistent with the literature that provides evidence for a strong and graded association between the severity of AGI and adverse outcomes in critically ill patients. We also observed that increasing AGI severity was associated with significantly higher mortality rates, which is consistent with the previous meta-analytic results (Morris et al., 2023). The pooled data from the meta-analysis revealed that critically ill patients with AGI grade IIIIV were at almost double the risk of death as those with less severe damage (risk ratio ≈ 1.86; 95% CI: 1.48–2.34) (Blaser et al., 2021). In our cohort, the 28-day mortality rate was 39.4% for AGI III–IV, 12.3% for AGI III, and 4.7% for the control group (p < 0.001), thus, the mortality gradient that was considerably higher in the studies that were recalled by our results is observed again. In a retrospective cohort study of elderly ICU patients suffering from gram-positive bloodstream infections, the occurrence of severe AGI was identified as being associated with death within 30 days. The adjusted hazard ratios for 30-day mortality were as high as 6.89 (95% CI: 2.34–20.29) for grades III-IV, which highlights an association with prognostic importance of AGI classification in severely ill patients (Li et al., 2023). Our findings in terms of severity scores closely mirror the existing body of evidence. The current study documented a substantial rise in both APACHE II and SOFA scores with the progression of AGI. In particular, the average APACHE II scores were 22.8 ± 5.6 and SOFA scores 10.4 ± 3.3 in AGI III, IV, as opposed to 14.6 ± 4.3 and 6.3± 2.1, respectively, in AGI I, II. Several previous observational studies have also reported a similar trend, i.e., higher SOFA scores in AGI patients, which in turn are associated with a higher risk of death. This suggests systemic organ dysfunction as a potential contributing factor in the deteriorating cycle of gastrointestinal injury. The extensive multicenter cohort has revealed that the combined use of AGI grades, APACHE II, and SOFA is associated with improved mortality prediction than when any of the scores is used alone. The results of this study also highlight important potential confounding factors that may influence both AGI severity and gut microbiota composition, including antibiotic exposure, proton pump inhibitor (PPI) use, duration of parenteral nutrition, ICU length of stay, and comorbidity burden, all of which were more prevalent in severe AGI cases and may be associated with observed microbiome alterations and outcomes (Liu et al., 2022). In our study, we revealed that feeding intolerance and the requirement of mechanical support were more frequently observed in individuals with a severe AGI condition. These findings agree with those obtained in the same groups of patients, where severely ill COVID, 19 patients with AGI III–IV have been associated with longer mechanical ventilation (22 days vs. 16 days, P = 0.002) and higher mortality (58% vs. 28%; HR = 2.68, 95% CI: 1.69–4.25) as compared to lower AGI grades. Such an example illustrates the physiological loop between gastrointestinal dysfunction, systemic inflammation, and prolonged organ support dependency in critical illness (Shen et al., 2024). Our study also demonstrates that old age and the presence of comorbidities (e.g., hypertension, diabetes, chronic kidney disease) were associated with risk factors for severe AGI and resultant negative outcomes. This resembles what has been observed in big ICU cohorts, where aged people, those with less organ reserve, and a high number of health issues are more likely to experience worsening organ dysfunction, particularly in the gastrointestinal system. For example, age, related decline in the intestinal barrier, and lower physiological resistance may be associated higher AGI severity and death, as shown by comparative clinical studies (Heyland et al., 2021). The patient outcomes we recorded, long ICU and hospital stays, a greater need for mechanical ventilation and vasopressors, as well as increased mortality rates that tracked higher AGI grades, are in line with worldwide ICU data that recognize gastrointestinal dysfunction as a marker of the severity of the primary critical condition and a worse prognosis. Several prospective observational studies have found that scores of gastrointestinal dysfunction (e.g., Gastrointestinal Dysfunction Score) and AGI grades have statistically significant relationships with 28-day mortality and ICU length of stay. Mortality prediction area under the curve (AUC) values becomes similar to those of APACHE II and SOFA when combined in composite models (Fan and Lee, 2021). The findings of this research support the therapeutic importance of standardized AGI grading as proposed by ESICM. Importantly, although formal stratified or sensitivity analyses were not performed in the present study, the observed consistency of associations across clinical subgroups (e.g., antibiotic-exposed vs. non-exposed patients) supports the robustness of our findings, and future studies should further explore stratified effects to strengthen causal inference. Our findings in **Table 2** show that patients with acute gastrointestinal injury (AGI) exhibited significant variations in gastrointestinal symptoms, nutritional parameters, and gut microbiota composition, which depended on the severity of the condition. These results not only confirm the data obtained from the previous research in the critically ill populations but also extend them. The extremely low number of clinical manifestations of gastrointestinal dysfunction feeding intolerance (78.9% in AGI III–IV vs. 35.5% in AGI III and 4.0% in controls), gastric residual volumes, diarrhea, abdominal distension, gastrointestinal bleeding, and paralytic ileus has been identified in the present research as both an indicator and a facilitator of severe systemic illness, which is consistent with the source cited previously. As an example, a prospective cohort study by Heyland et al. found that up to 70% of patients with severe gastrointestinal dysfunction experienced feeding intolerance, which, in turn, was associated with organ failure and death; their results are very similar to ours in the AGI III–IV group (Nakane et al., 2021). Moreover, the occurrence of small intestinal bacterial overgrowth and endotoxemia has been linked with diaphragmatic and stomach dysmotility in severely ill patients, thus supporting an association with the clinical importance of the gastrointestinal symptoms revealed in our group (Nakane et al., 2021).

Our data reveal that the start of enteral nutrition was significantly delayed (4.6± 1.8 days in AGI III–IV) and that the rate of enteral interruption was very high (60.6%) in AGI III–IV as compared to AGI III and the control group, as well as that the caloric adequacy was reduced (51.2 ± 14.3%). The same patterns have been reported by Fan and Lee; in their study, delayed or interrupted enteral feeding in critically ill patients was associated with increased infection rates and decreased gut barrier function (Salameh et al., 2023). In inadequately fed patients, caloric intake was often only 40-60% of the target. The high rate of parenteral nutrition required (76.7% in AGI III–IV) that we have observed is in agreement with these studies and supports the association between severe AGI impairing nutrient delivery and utilization, that linked with catabolism and immunosuppression to be aggravated. Our biochemical markers in this investigation showed that serum albumin, prealbumin, and citrulline levels were significantly lowered in AGI III–IV, while the level of intestinal fatty acid-binding protein (I-FABP) was elevated. These data echo the work of other researchers who have shown that citrulline, a metabolite specific to enterocytes, is a sensitive biomarker of the number and the functional capacity of enterocytes, and that the cut-off value of less than 20 mol/L is indicative of severe mucosal damage and poor prognosis in critical illness. Similar to that, Nakane et al. also discovered that low levels of albumin and prealbumin are associated with gastrointestinal hypomotility and bacterial translocation (Akhtar et al., 2022). This is in agreement with our study that severe AGI is not only a manifestation of functional dysmotility but also a sign of serious mucosal and nutritional problems. The gut microbiota changes reported in this work, i.e., significantly decreased alpha diversity (e.g., Shannon index 2.71 ± 0.48 for AGI III–IV vs. 4.28 ± 0.61 for controls, p < 0.001) and changes in dominant phyla, are in line with a large and growing number of publications that confirm dysbiosis associated with critical disease. Our findings on the reduction of microbial richness and diversity are comparable with what Salameh et al. reported, where critically ill patients had their Shannon indices ranging from 2.0 to 3.0, which indicates their ecosystems were highly disturbed as compared to healthy controls (who normally had indices above 4.0). Our cohort’s increase in Proteobacteria (33.5 ± 9.3% in severe AGI) is consistent with *Proteobacteria-*centric ICU microbiome studies, which imply that when the proportion of Proteobacteria exceeds 30%, it is associated with inflammation, endotoxin release, and negative clinical outcomes, among which are sepsis and multi-organ dysfunction (Ojima et al., 2022). At the genus level, the excessive presence of opportunistic taxa such as *Escherichia* and *Shigella* (18.9 ± 6.8%) and *Klebsiella* (11.6 ± 4.9%) in AGI III–IV patients confirms the previous studies that have found that critical illness and the use of broad-spectrum antibiotics are associated with proliferation of *Enterobacteriaceae* and other pathobionts. These alterations have been associated with increased intestinal permeability, translocation of bacterial products, and immune system activation throughout the body, which exacerbates organ dysfunction. Conversely, the disappearance of beneficial commensals such as *Lactobacillus*, *Bifidobacterium*, and *Faecalibacterium* is consistent with what Akhtar et al. found in their clinical studies: that the loss of these taxa results in decreased production of short-chain fatty acids and mucosal anti-inflammatory signaling. This may perpetuate the cycle of gut barrier failure and systemic inflammation (Shimizu et al., 2021). The severity of AGI is reflected in associations with the patient, related variables, clinical condition, treatment, nutritional status, and profound gut microbial dysbiosis, as quantitatively demonstrated by the current retrospective study. Notably, in comparison to other investigations, the magnitude and direction of these correlations exhibit significant consistency, while also providing more precise effect estimates. Age was identified as an independent risk factor for severe AGI in our population, with each additional year being associated with the risks by 4% (OR = 1.04). This impact size is similar to what Ojima et al. found, which was a 3–5% rise in gastrointestinal dysfunction for every year of age in critically sick patients (Liu et al., 2023). Shimizu et al. also found that patients over 60 years old had much less microbial diversity, with a mean Shannon index drop of about 0.3 to 0.5 units. This is similar to the age-related drop we found in our regression model (β = –0.012 per year) (Umemoto et al., 2023). Scores for the severity of sickness had the strongest links to AGI advancement and microbiome disturbance. Because several treatment-related variables may be influenced by baseline illness severity, additional adjusted analyses were conducted to control for major confounders including age, sex, and APACHE II score. Following adjustment, the associations between antibiotic exposure, proton pump inhibitor use, interruption of enteral nutrition, and microbial dysbiosis remained significant, supporting their independent association with AGI progression beyond illness severity alone.

In our analysis, each one-point rise in the APACHE II score was associated with the odds of severe AGI by 21% (OR = 1.21), which is quite similar to what Liu et al. found, where the ORs for gastrointestinal failure per APACHE II point ranged from 1.18 to 1.25. The SOFA score revealed a stronger association (OR = 1.35 per point) (Haak et al., 2021), which is very similar to the results of Umemoto et al., who showed that patients with SOFA 10 had a two, to threefold increased risk of intestinal dysfunction (Koyyada, 2021). On the other hand, high APACHE II and SOFA scores in our group also showed significant microbial changes, in particular, a 2.02.8% increase in Proteobacteria per score point and a 0.080.10 decrease in the Shannon index, which are very close to the values reported in the ICU microbiome study by Haak et al (Reintam Blaser et al., 2023). In our study, being exposed to antibiotics was linked to a 2.47-fold higher chance of having severe AGI. This is similar to what other ICU studies have found, which showed that the odds of having gastrointestinal problems after taking broad-spectrum antibiotics were between 2.0 and 3.0. Furthermore, the corresponding increase in Proteobacteria abundance (+4.5%) and the decrease in Shannon diversity (β = –0.21) align with the dysbiosis patterns identified by Koyyada, who noted Proteobacteria proportions exceeding 30% and Shannon indices below 3.0 in patients with significant antibiotic exposure. Likewise, the utilization of proton pump inhibitors increased the probability of severe AGI (OR = 2.03), consistent with meta-analyses indicating ORs of 1.8–2.5 for PPI-associated gut dysbiosis and enteric complications in hospitalized patients (Castaño-Jiménez et al., 2025).

Nutritional parameters had some of the most substantial effect sizes. Stopping enteral nutrition was associated with a higher chance of severe AGI by more than three times (OR = 3.12), which is more than the two times risk that Reintam Blaser et al. found for feeding intolerance and gastrointestinal damage in ICU patients (Reintam Blaser et al., 2023). The microbiota changes we saw in our study, Shannon index dropping by 0.26 units and Proteobacteria growing by 5.8%, are consistent with associations reported in experimental and clinical studies that not feeding someone through their mouth leads to pathogenic overgrowth and loss of helpful anaerobes. On the other hand, higher serum albumin levels were quite protective (OR = 0.88 per g/L), which is similar to what earlier studies found: people with low albumin levels (<30 g/L) had two to three times more gastrointestinal problems. The positive correlation between albumin and microbial diversity (β = 0.12) corroborates previous findings that associate sufficient nutritional and inflammatory conditions with the maintenance of gut microbial homeostasis (Reintam Blaser et al., 2023). Lastly, gut microbiome indices were independent indicators of AGI severity. A greater Shannon index lowered the probability of severe AGI by 59% (OR = 0.41). This is quite similar to what Castaño-Jiménez et al. found, which was a 50–60% lower risk of gastrointestinal failure in patients with intact microbial diversity. On the other hand, every 1% rise in Proteobacteria (OR = 1.09) and *Escherichia–Shigella* (OR = 1.12) was associated with increased AGI severity, akin to previous findings indicating that Proteobacteria predominance (>30%) is linked with elevated death and organ failure rates (Castaño-Jiménez et al., 2025).

### 4.1 Strengths and limitations

This study has several notable strengths, including a large multicenter sample and the integrated evaluation of clinical, nutritional, and gut microbiota characteristics across different levels of AGI severity. However, certain limitations should be acknowledged. The retrospective design restricts causal inference and may be susceptible to residual confounding. The use of convenience sampling introduces potential selection bias and may limit the generalizability of the findings to broader populations. In addition, the relatively short four-month data collection period may reduce temporal representativeness across seasons and years. Furthermore, the predominance of male participants, inherent to the retrospective dataset, may introduce sex-related bias despite adjustment in multivariate analyses. Although major confounding factors were adjusted for in multivariate analyses, residual confounding from unmeasured clinical variables cannot be completely excluded.

## 5 Conclusion

Severe AGI was strongly associated with older age, a higher number of comorbidities, a more severe critical condition, and more aggressive treatments in the ICU. The level of severe AGI was associated with extreme malnutrition, significantly delayed and disrupted enteral nutrition, and reliance on parenteral feeding, thus being accompanied by very low serum albumin, prealbumin, and citrulline levels. Gut microbiota analysis revealed substantial dysbiosis that was characterized by reduced alpha diversity and overgrowth of pathogenic taxa such as Proteobacteria and *Escherichia, Shigella*, and a deficiency of beneficial genera such as *Lactobacillus*, *Bifidobacterium*, and *Faecalibacterium*. Moreover, multivariate analyses confirmed that clinical severity, nutritional status, and specific microbiota features were independently associated with the progression of AGI, thus suggesting their interrelated role in the pathogenesis of the disease. The results highlight the potential importance of early nutritional intervention, the judicious use of antibiotics and proton pump inhibitors, and the implementation of microbiota-restoring therapies as effective strategies to potentially prevent the development of severe AGI and may contribute to improving the clinical outcomes of critically ill patients. Additionally, strategies such as early risk screening, individualized nutrition support, targeted probiotic or synbiotic supplementation, and microbiome-based therapeutic approaches may offer promising supportive avenues for both prevention and treatment of AGI.

## Data Availability

The original contributions presented in the study are included in the article/supplementary material. Further inquiries can be directed to the corresponding author.
